# Contribution of the Oral and Gastrointestinal Microbiomes to Bloodstream Infections in Leukemia Patients

**DOI:** 10.1128/spectrum.00415-23

**Published:** 2023-04-06

**Authors:** Stephanie McMahon, Pranoti Sahasrabhojane, Jiwoong Kim, Samantha Franklin, Chia-Chi Chang, Robert R. Jenq, Andrew E. Hillhouse, Samuel A. Shelburne, Jessica Galloway-Peña

**Affiliations:** a Interdisciplinary Genetics Program, Texas A&M University, College Station, Texas, USA; b Department of Infectious Diseases, Infection Control, and Employee Health, MD Anderson Cancer Center, Houston, Texas, USA; c Department of Bioinformatics and Harold C. Simmons Comprehensive Cancer Center, University of Texas Southwestern Medical Center, Dallas, Texas, USA; d Department of Genomic Medicine, MD Anderson Cancer Center, Houston, Texas, USA; e Department of Veterinary Pathobiology, Texas A&M University, College Station, Texas, USA; f Texas A&M Institute for Genome Sciences & Society, Texas A&M University, College Station, Texas, USA; Lerner Research Institute

**Keywords:** oral microbiome, intestinal domination, bacteremia, leukemia, colonization

## Abstract

Bloodstream infections (BSIs) pose a significant mortality risk for acute myeloid leukemia (AML) patients. It has been previously reported that intestinal domination (>30% relative abundance [RA] attributed to a single taxon) with the infecting taxa often precedes BSI in stem cell transplant patients. Using 16S rRNA amplicon sequencing, we analyzed oral and stool samples from 63 AML patients with BSIs to determine the correlation between the infectious agent and microbiome composition. Whole-genome sequencing and antimicrobial susceptibilities were performed on all BSI isolates. Species-level detection of the infectious agent and presence of antibiotic resistance determinants in the stool (*bla*_CTX-M-15_, *bla*_CTX-M-14_, *cfrA*, and *vanA*) were confirmed via digital droplet PCR (ddPCR). Individuals with Escherichia coli (stool *P* < 0.001), Pseudomonas aeruginosa (oral *P = *0.004, stool *P < *0.001), and viridans group streptococci (VGS) (oral *P = *0.001) bacteremia had a significantly higher relative abundance of those respective genera than other BSI patients, which appeared to be site specific. Although 78% of patients showed presence of the infectious genera in the stool and/or saliva, only 7 exhibited microbiome domination. ddPCR confirmed species specificity of the 16S data and detected the antibiotic resistance determinants found in the BSI isolates within concurrent stools. Although gastrointestinal (GI) domination by an infecting organism was not present at the time of most BSIs in AML, the pathogens, along with AMR elements, were detectable in the majority of patients. Thus, rapid genetic assessment of oral and stool samples for the presence of potential pathogens and AMR determinants might inform personalized therapeutic approaches in immunocompromised patients with suspected infection.

**IMPORTANCE** A major cause of mortality in hematologic malignancy patients is BSI. Previous studies have demonstrated that bacterial translocation from the GI microbiome is a major source of BSIs and is often preceded by increased levels of the infectious taxa in the GI (>30% abundance by 16S rRNA sequencing). In this study, we sought to better understand how domination and abundance levels of the oral and gut microbiome relate to bacteremia occurrence in acute myeloid leukemia patients. We conclude that analyses of both oral and stool samples can help identify BSI and antimicrobial resistance determinants, thus potentially improving the timing and tailoring of antibiotic treatment strategies for high-risk patients.

## INTRODUCTION

Acute myeloid leukemia (AML) is the most common acute leukemia affecting adults, and patients are typically treated with extensive cytotoxic chemotherapy. Due to the nature of the malignancy, AML patients have severely compromised immune systems in addition to degradation of the gut mucosa attributable to the cytotoxic chemotherapy (1). The combination of weakened mucosal lining and decreased immune response renders AML patients at high risk for infection. Approximately 20 to 40% of AML patients undergoing treatment will develop a microbiologically documented infection (2–5). Specifically, AML patients are at the highest risk of contracting a bloodstream infection (BSI) compared to patients with other hematologic malignancies (6, 7). It is presumed that bacteremia without a focus arises from organisms that typically colonize the gastrointestinal tract (GI), gaining access to the bloodstream due to mucosal damage and/or immune deficiency (8).

Along with chemotherapy, antibiotic-induced microbial dysbiosis can contribute to an increased infection rate (9). AML patients can receive up to an average of 6 antibiotics during a 28-day inpatient stay following induction chemotherapy, which commonly includes prophylactic antibiotic treatment prior to chemotherapy initiation, empirical therapy upon onset of neutropenic fever, and treatment antibiotics based on positive microbiological cultures (2, 10). Antibiotic disruption of the intestinal microbiota can promote gut inflammation and impact the composition and function of the microbiome by depleting beneficial commensal bacteria and increasing the risk of intestinal establishment with antibiotic-resistant bacteria (11–15). Intestinal domination by bacteria causing infection has been shown to predict BSI in cohorts such as stem cell transplant patients, postoperative surgical patients, and patients with other hematologic malignancies (16–19). Although associations exist between the presence of specific bacteria in the gut and BSIs, not all intestinal domination events lead to a matching BSI (6). Moreover, it was recently reported that nonenteric pathogens, such as Pseudomonas aeruginosa and Staphylococcus epidermidis, are found also in the gut microbiota of heme malignancy patients preceding bloodstream infection, thereby challenging the existing informal dogma that nonenteric infections originate from environmental or skin sources (20). Thus, the relationship between intestinal domination events and bacteremia in AML patients remains unclear.

Given that monitoring of the gut microbiome might be useful in identifying patients at high risk of bacteremia, in this study, we sought to better understand how domination and abundance levels in the fecal microbiome relate to bacteremia occurrence in AML patients (17, 21, 22). Conflicting published data have raised the question of whether intestinal domination is indeed an important event for bacteremia to occur, as it had been previously suggested by other studies in mice and allogeneic hematopoietic cell transplantation patients (allo-HCT) (6, 10, 16, 20, 22–25). These data, combined with the high burden of nonenteric infectious disease in AML patients, led us to hypothesize that if the gastrointestinal tract is not dominated, then perhaps the oral cavity is the major place of burden. Thus, in addition to analyzing the stool, we also aimed to determine if analyzing the oral cavity would generate additional insights. Specifically, we characterized bacteremia-causing isolates, stool, and oral samples from a cohort of AML patients, with the overall goal of determining if rapid analyses of both oral and stool samples can help identify BSI and antimicrobial resistance determinants.

## RESULTS

### Phylogenetic characterization of bacteremia isolates.

In order to understand the epidemiology of the etiological agents causing BSI among AML patients at our hospital and their association with patient microbiome characteristics, we executed a multisite sampling study of bloodstream isolates, oral, and stool samples collected from a cohort of AML patients with bacteremia. AML patients positive for a bacterial BSI were identified through collaboration with the clinical microbiology laboratory from September 2014 through January 2019. Of note, these patients were a random convenience subsampling of AML patients based on staff availability and patient consent. Whole-genome sequencing (WGS) was performed on the bloodstream isolates. Of the organisms causing BSI among the 63 patients enrolled, viridans group streptococci (VGS) represented the highest number of infections at 22% of the 64 positive blood cultures that were analyzed (Table 1). The other species that comprised the etiological agents of bacteremia within these patients were Escherichia coli (20%), Staphylococcus epidermidis (19%), Pseudomonas aeruginosa (16%), *Enterococcus* spp. (E. faecium and E. faecalis) (10%), Staphylococcus aureus (8%), and Klebsiella pneumoniae (6%). Multilocus sequence typing information derived from whole genome sequencing data is shown in Table 1. For three of the causative agents (*Enterococcus* spp., S. aureus, and S. epidermidis), most isolates were from one or two clonal sequence types (STs), as opposed to multiple different community-derived or hospital-associated sequence types as was seen in P. aeruginosa and E. coli (26–33). The predominant ST for S. aureus and S. epidermidis was ST5, whereas the predominant clone for E. faecalis was ST6 and ST17 for E. faecium (Table 1). To better understand the relevance of our collected subset of AML patient samples to the broader leukemia population, we determined the etiology of all positive blood cultures in the years 2019 to 2021 from the electronic medical record. Similar to the distribution of infectious agents among our subsampling of AML patients who contracted bacteremia between 2014 and 2019, 52% of unique positive blood cultures tested for susceptibilities from leukemia patients at MD Anderson Cancer Center (MDACC) (2019 to 2021) were caused by classically nonenteric bacteria, most notably S. epidermidis (19.2%, *n* = 136/703), viridans group streptococci (12.4%, *n* = 87/703), P. aeruginosa (8.1%, *n* = 57/703), S. aureus (4.9%, *n* = 34/703), and Stenotrophomonas maltophilia (4.4%, *n* = 31/703).

**TABLE 1:** Frequency of etiological agents for bloodstream infection in patients with acute leukemia undergoing induction chemotherapy

Bacteria isolated from blood culture	Frequency of infection (%)	Sequence types identified^*a*^
Viridans group streptococci	22 (*n* = 14)	Streptococcus mitis (*n* = 6), Streptococcus sanguinis (*n* = 1), Streptococcus parasanguinis (*n* = 2), Streptococcus salivarius (*n* = 1), Streptococcus oralis ST1 (*n* = 1), S. oralis ST34 (*n* = 1), S. oralis ST75 (*n* = 1), S. oralis ST21 (*n* = 1)
Escherichia coli	20 (*n* = 13)	ST131 (*n* = 5), ST648 (*n* = 2), ST405 (*n* = 4), ST156 (*n* = 1), ST1193 (*n* = 1)
Staphylococcus epidermidis	19 (*n* = 12)	ST369 (*n* = 1), ST7 (*n* = 1), ST84 (*n* = 2), ST7 (*n* = 8)
Pseudomonas aeruginosa	16 (*n* = 10)	ST179 (*n* = 1), ST2274 (*n* = 1), ST253 (*n* = 1), ST17 (*n* = 1), ST1710 (*n* = 1), ST167 (*n* = 1), ST111 (*n* = 1), ST2629 (*n* = 1), ST235 (*n* = 1), ST2613 (*n* = 1)
Staphylococcus aureus	8 (*n* = 5)	ST8 (*n* = 1), ST231 (*n* = 1), ST72 (*n* = 1), ST5 (*n* = 2)
Klebsiella pneumoniae	6 (*n* = 4)	ST3292 (*n* = 1), ST1083 (*n* = 1), ST45 (*n* = 1), ST307 (*n* = 1)
Enterococcus faecium	5 (*n* = 3)	ST17 (*n* = 2), ST664 (*n* = 1)
Enterococcus faecalis	5 (*n* = 3)	ST6 (*n* = 3)
Total	100 (*n* = 64)	

a Multilocus sequence types are not available in PubMLST for S. mitis, S. sanguinis, S. parasanguinis, and S. salivarius. The numbers of infections identified by species for viridans group streptococci are provided instead.

### Enrichment of specific taxa that caused infection in oral and stool sites.

When analyzing both the oral and the stool microbiome, a critical finding was at the time of bacteremia when we only identified 7 domination events with the infecting genera in either the oral or stool of our 64 bacteremia episodes (Table 2). Only 1 of 13 stool samples from patients with E. coli bacteremia showed domination levels (>30% of 16S rRNA reads) despite 9 of 13 showing presence of Escherichia by 16S sequencing. Only 1 of 6 fecal samples from patients with BSI attributed to *Enterococcus* spp. reached domination levels, even with 5 of 6 stool samples showing presence of *Enterococcus*. Two out of 10 oral samples from P. aeruginosa BSI showed domination compared to the 5 out of 10 identified to have presence of the Pseudomonas genera. Two of 12 stool samples from patients with S. epidermidis BSI showed domination, although 6 of 12 confirmed the presence of Staphylococcus genera. Out of 14 samples, only 1 VGS stool sample reached domination levels. Five of 14 showed presence in oral samples, and 10 of 14 showed presence in stool samples. Neither S. aureus nor K. pneumoniae showed domination in any of their samples.

**TABLE 2:** Representation of 16S rRNA abundance levels of infectious genera in accordance with the etiological agent of bloodstream infection

Infecting species	Presence^*a*^ of infecting genera (no. detected/total no. of samples) in:		Colonization^*b*^ with infecting genera (no. detected/total no. of samples) in:		Domination^*c*^ by infecting genera (no. detected/total no. of samples) in:		Avg relative abundance in:	
	Oral	Stool	Oral	Stool	Oral	Stool	Oral	Stool
E. faecalis	2/3	2/3	0/3	1/3	0/3	0/3	0.0182	0.2266
E. faecium	2/3	3/3	1/3	3/3	0/3	1/3	0.0281	0.1911
E. coli	4/13	9/13	0/13	3/13	0/13	1/13	0.0004	0.0799
K. pneumoniae	0/4	1/4	0/4	0/4	0/4	0/4	0.0000	0.0001
P. aeruginosa	5/10	3/10	3/10	0/10	2/10	0/10	0.1840	0.0007
S. aureus	4/5	3/5	0/5	1/5	0/5	0/5	0.0118	0.0238
S. epidermidis	7/12	6/12	1/12	3/12	0/12	2/12	0.0159	0.1431
VGS^*d*^	5/14	10/14	0/14	2/14	0/14	1/14	0.0227	0.0049

a Presence of infecting genera defined as >0.0001 relative abundance by 16S rRNA sequencing.

b Colonization with infecting genera defined as >0.03 relative abundance by 16S rRNA sequencing.

c Domination by infecting genera defined as >0.30 relative abundance by 16S rRNA sequencing.

d VGS, viridans group streptococci.

**FIG 1:**
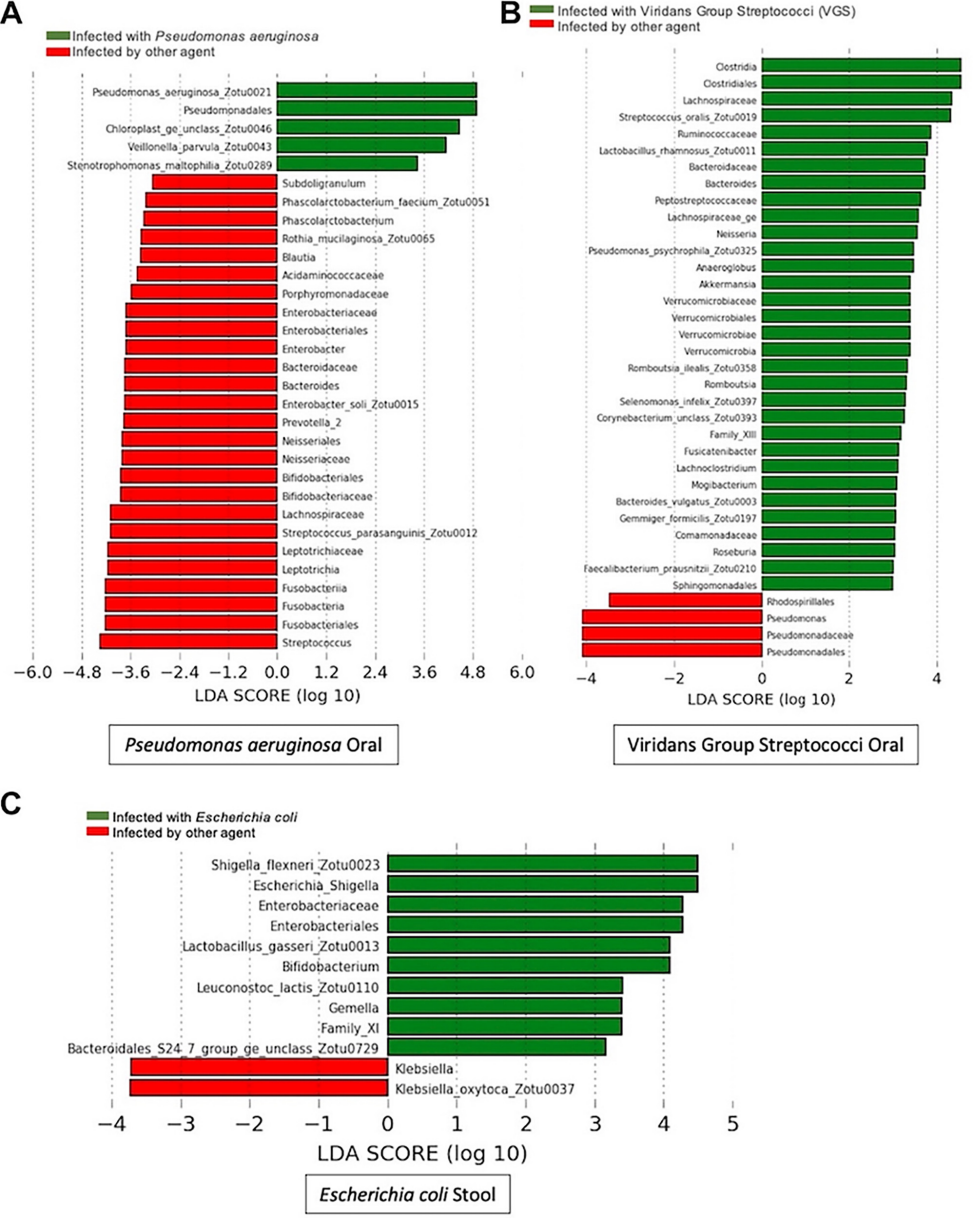
Linear discriminant analysis for effect size (LEfSe) analysis shows enrichment of taxa that cause respective infections. LEfSe was performed on stool and oral samples for each infectious agent designated to determine differences in the microbiome by site and infectious species. Organisms with a green bar are enriched in patients with the designated infection, while organisms with a red bar are enriched in patients who do not have that designated infection. Only the graphs in which the site was enriched with the etiological agent are shown.

Despite the lack of domination events, we wanted to establish whether sampling of the oral or stool microbiome could assist with identifying the infecting bacteria in a patient with bacteremia. We performed linear discriminant analysis for effect size (LEfSe) analysis to resolve if there was genus-specific enrichment in the oral cavity or stool microbiome with the same genera which caused the BSI. We analyzed each causative agent individually, comparing the enrichment of specific microbiota in people who had that infection compared to people who did not have an infection caused by that agent (Fig. 1). Interestingly, we only identified specific genera enrichment in the microbiome for three of the seven species which caused infection. Pseudomonas was enriched in both the oral cavity and stool among patients exhibiting P. aeruginosa BSIs, Escherichia enrichment was observed in fecal samples among patients with E. coli bacteremia, and Streptococcus enrichment was observed in the oral cavity among patients with BSIs due to viridans group streptococci (Fig. 1A to C). Our LefSe analysis of patients with BSIs caused by *Enterococcus* spp., K. pneumoniae, S. epidermidis, and S. aureus showed differential enrichment of other taxa, but not of the genera that caused the bacteremia (see Fig. S1 to S4 in the supplemental material). To delve further, we used Mann-Whitney testing to compare the distribution of the infecting taxa within oral and stool samples. We saw results consistent with our LEfSe analysis in that there were only significantly higher abundances of the infecting genera among patients with P. aeruginosa (oral and stool), E. coli (stool), and VGS (oral) bacteremia (Fig. S5).

Our lack of identification of domination in our single time point analysis caused us to analyze previously generated longitudinal microbiome data from an AML cohort. It is possible that our samples may have been collected after a critical point for domination, and thus, we missed the potential window to observe this occurrence in the GI tract. To determine that this was not due to a missed time window prior to infection, we took longitudinal data available from our previously published data set (BioProject accession no. PRJNA352060 and PRJNA526551) (24). Out of 15 patients who contracted a BSI during induction chemotherapy in those studies, only 4 patients had a relative abundance of 30% or greater of their infectious agent in their oral or stool in any of the 30 days prior to their BSI. Domination was seen for Escherichia, *Enterococcus*, and Pseudomonas in the stool and Pseudomonas in the oral. So, although domination occurred, it occurred in a considerably low percentage (12.5%) of AML patients contracting bacteremia. This indicates that intestinal domination is not necessarily an indicator of bacteremia in AML patients.

### Abundances differ based on site and species specificity.

**FIG 2:**
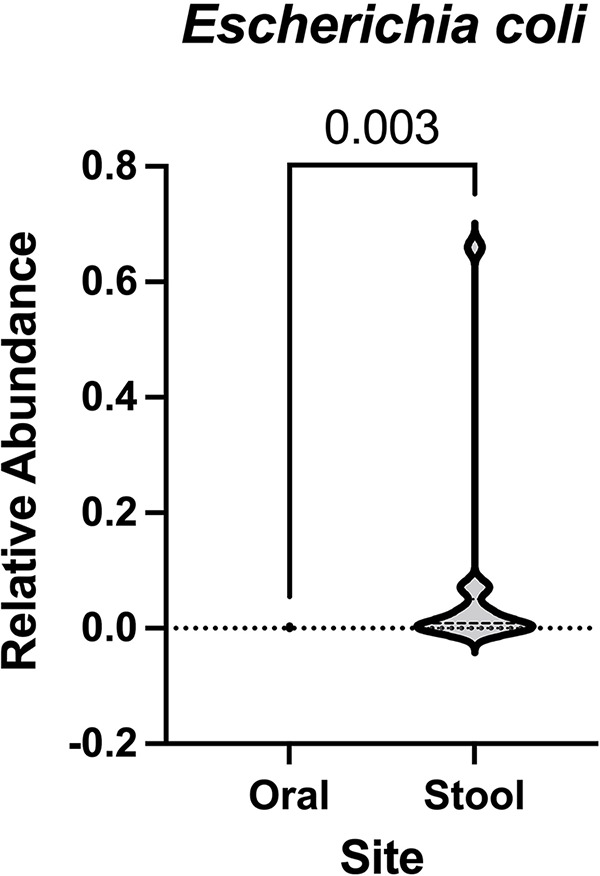
Only patients with Escherichia coli infections showed differences in relative abundance between their stool and oral samples. Wilcoxon testing was performed on infectious agents of interest to determine if patients showed differing levels of abundance of their etiological agent between their stool and oral microbiomes. This was done by grouping patients by each infectious agent and plotting the relative abundance of the infectious agent in stool samples versus the relative abundance of the infectious agent in oral samples. The only set of patients that showed a statistically significant difference between samples were patients with E. coli infections.

Given that many of the isolates causing bacteremia in our cohort are thought to originate from either the oropharynx or the intestines, we hypothesized that enrichment would differ by site based on the pathogenesis of each infection. Thus, we next sought to identify which site, oral or stool, contained a higher relative abundance of infecting pathogens for each patient with bacteremia caused by a specific agent. To this end, via Wilcoxon testing, we compared oral and stool abundances among patients who contracted a specific bacterial infection. We found that only the relative abundance of Escherichia differed significantly between the oral cavity and fecal samples among patients who contracted an E. coli infection. In patients contracting E. coli bloodstream infections, Escherichia was significantly more abundant in the stool than in the oral microbiome (*P* = 0.003) (Fig. 2). We did not find a statistically significant difference between the oral and stool relative abundances for patients with infection with *Enterococcus* spp. (*P* = 0.438), K. pneumoniae (*P* ≥ 0.999), P. aeruginosa (*P* = 0.125), S. aureus (*P* = 0.875), S. epidermidis (*P* = 0.078), or Streptococcus spp. (*P* = 0.275) (Fig. S6).

### Digital droplet PCR to confirm abundances at the species level.

**FIG 3:**
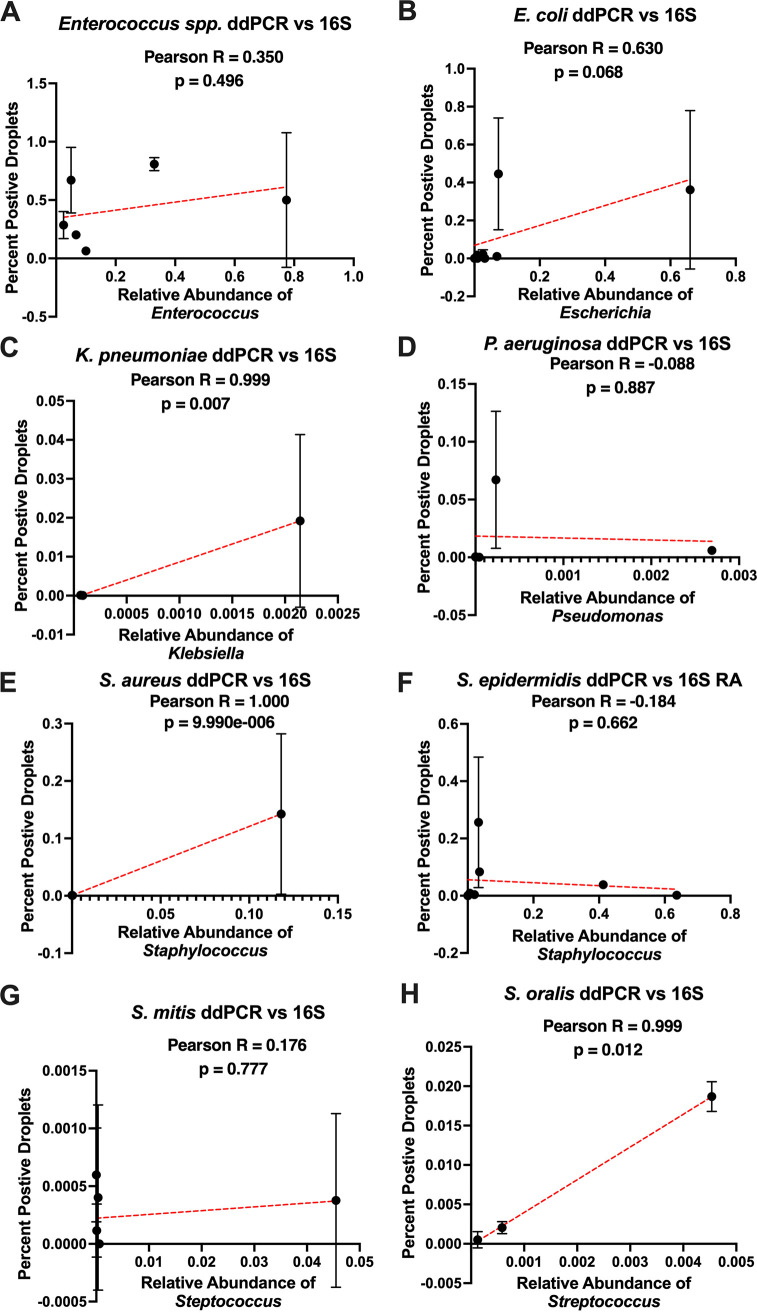
Graphical depiction of percent positive droplets by ddPCR compared to relative abundance via 16S rRNA sequencing. Digital droplet PCR was performed on DNA extracted from stool samples of patients who were infected by Escherichia coli (A), *Enterococcus* spp. (B), Streptococcus mitis (C), Streptococcus oralis (D), Staphylococcus epidermidis (E), Klebsiella pneumoniae (F), Pseudomonas aeruginosa (G), and Staphylococcus aureus (H). ddPCR-positive percentages were determined by dividing the number of positive droplets by the total number of droplets. Those values were plotted against the relative abundance (RA) values gathered from 16S rRNA gene sequencing. The Pearson test was used to determine the correlation coefficient (*r*) and *P* values for all graphs. Simple linear regression was used to plot a line of best fit (red line) on each graph, where each dot represents an individual patient. A ROUT analysis was used to remove outliers from each data set prior to preforming the analysis.

The 16S gene amplicons often map to the genus level with high confidence approximately 90% of the time but are far less consistent when mapping to the species level (34). Although we assumed the 16S rRNA reads mapping to the genera of interest were coming from the species causing infection, they could potentially be from a different species within the same genera. Hence, we performed ddPCR targeting the specific infectious species causing BSI in stool samples which also had the presence of the infectious genera identified via 16S amplicon sequencing. We observed that, in general, the two methods trend positively or are mostly positively correlated with each other (Fig. 3). Each etiological agent of infection was investigated in depth to verify the agreement between the ddPCR and 16S rRNA V4 amplicon sequencing among stool samples (Table 3). Out of 44 tests, 31 total tests were in agreement, 24 positive by both methods, 7 negative by both methods, and 13 tests showed a disagreement. Cohen’s kappa value comparing the validity and reliability of ddPCR to 16S detection was 0.3093, indicating a moderate agreement between the two methods of detection. Overall, ddPCR detected the specific species causing BSI in 66% of stool samples tested. In comparison, 16S rRNA amplicon sequencing detected the genera causing BSI in 73% of the stool samples tested.

**TABLE 3:** Comparison of organism identification between 16S rRNA sequencing and digital droplet PCR

Etiological agent	No. of samples tested	No. positive by ddPCR	No. positive by 16S	No. positive by both
E. coli	9	7	8	6
*Enterococcus spp.*	6	6	5	5
K. pneumoniae	3	1	1	1
P. aeruginosa	6	4	2	2
S. aureus	4	1	2	1
S. epidermidis	8	7	6	6
S. mitis	5	1	5	1
S. oralis	3	2	3	2

### Digital droplet PCR confirms the presence of antibiotic resistance determinants in the stool that were present in bacteremia isolates.

**FIG 4:**
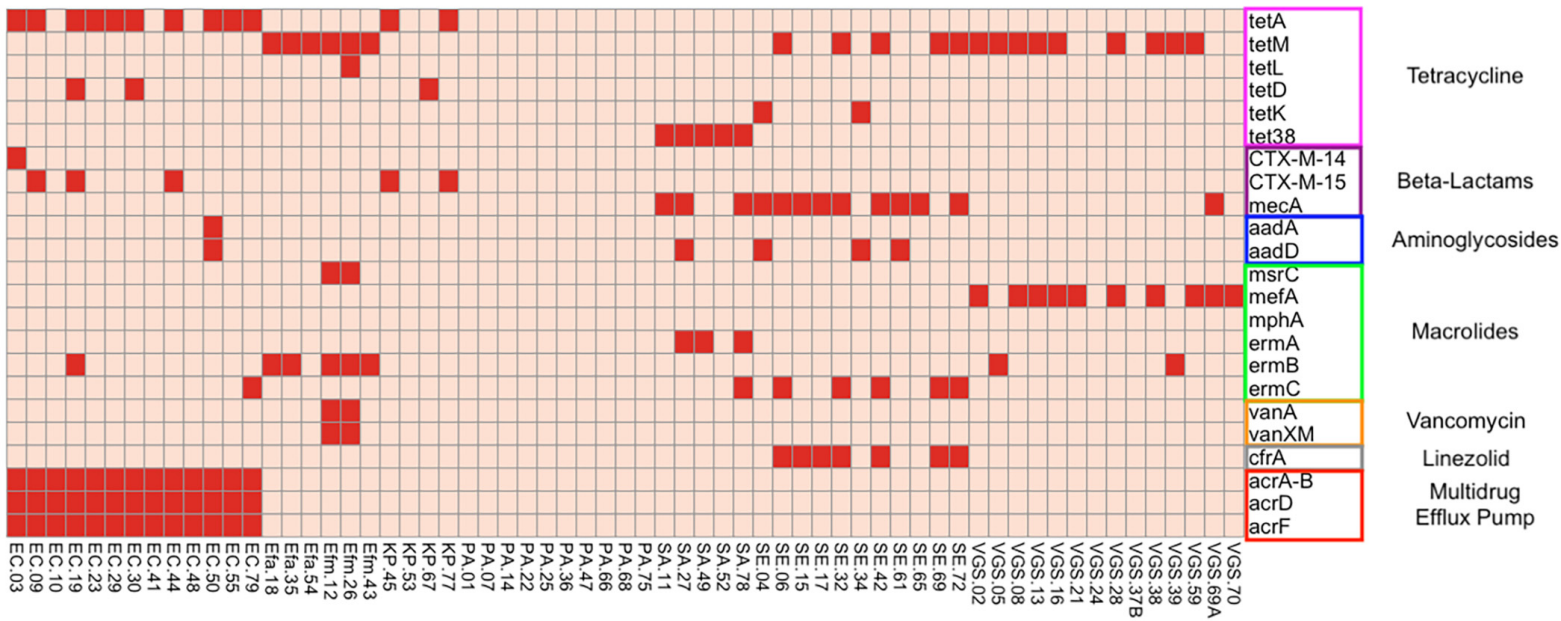
Presence of acquired resistance determinants among bloodstream isolates. A binary heatmap is shown based on the presence or absence of specific acquired resistance genes present among all infectious isolates. The *x* axis is organized alphabetically by species, and the *y* axis is organized according to the antibiotic resistance conferred by each gene. The heatmap is colored from pale red, indicating a gene is not present, to bright red, indicating that a gene is present in that isolate. Species are abbreviated as follows: Escherichia coli, EC; Enterococcus faecium, Efm; Enterococcus faecalis, Efa; Pseudomonas aeruginosa, PA; Klebsiella pneumoniae, KP; Staphylococcus aureus, SA; Staphylococcus epidermidis, SE; and viridans group streptococci, VGS.

To ideally guide antimicrobial administration, a genetic test would need to identify both the pathogen and antimicrobial resistance elements. Therefore, we sought to determine whether we could identify the genetic elements driving bloodstream isolate antimicrobial resistance in the stool samples of infected patients. First, WGS was performed on the BSI isolates, and protein sequences were identified using a custom pipeline built by merging the data of antibiotic resistance database (ARDB) and comprehensive antibiotic resistance database (CARD) (35; https://card.mcmaster.ca/). Figure 4 shows a binary heatmap for presence or absence of common chromosomal and acquired resistance elements. We then compared these data to the phenotypic antibiotic resistance profiles derived from patient electronic medical records (Fig. S7).

Among the antimicrobial resistance elements identified, the genes conferring linezolid, vancomycin, and β-lactam resistance are readily detectable via PCR methodology. When examining the K. pneumoniae isolates, 2 out of 4 contained genes such as *bla*_CTX-M-15_ and *bla*_CTX-M-14_ β-lactamases, which coincided with 50% being resistant. Out of 14 E. coli isolates, 4 contained *bla*_CTX-M-15_ or *bla*_CTX-M-14_ genes; however, 5 isolates were phenotypically resistant to 3rd-generation cephalosporins. When examining S. epidermidis whole-genome sequences, 7 of the 12 isolates contained the *cfrA* gene, a 23S rRNA methyltransferase conferring resistance to antibiotics such as linezolid. Interestingly, only 4 of the 6 *cfr*-containing isolates were deemed phenotypically linezolid resistant (1 was not tested). Two of 6 *Enterococcus* species isolates contained genes for vancomycin resistance (*vanA* and *vanXM*), but a third isolate was resistant to vancomycin, suggesting an additional genetic mechanism.

**FIG 5:**
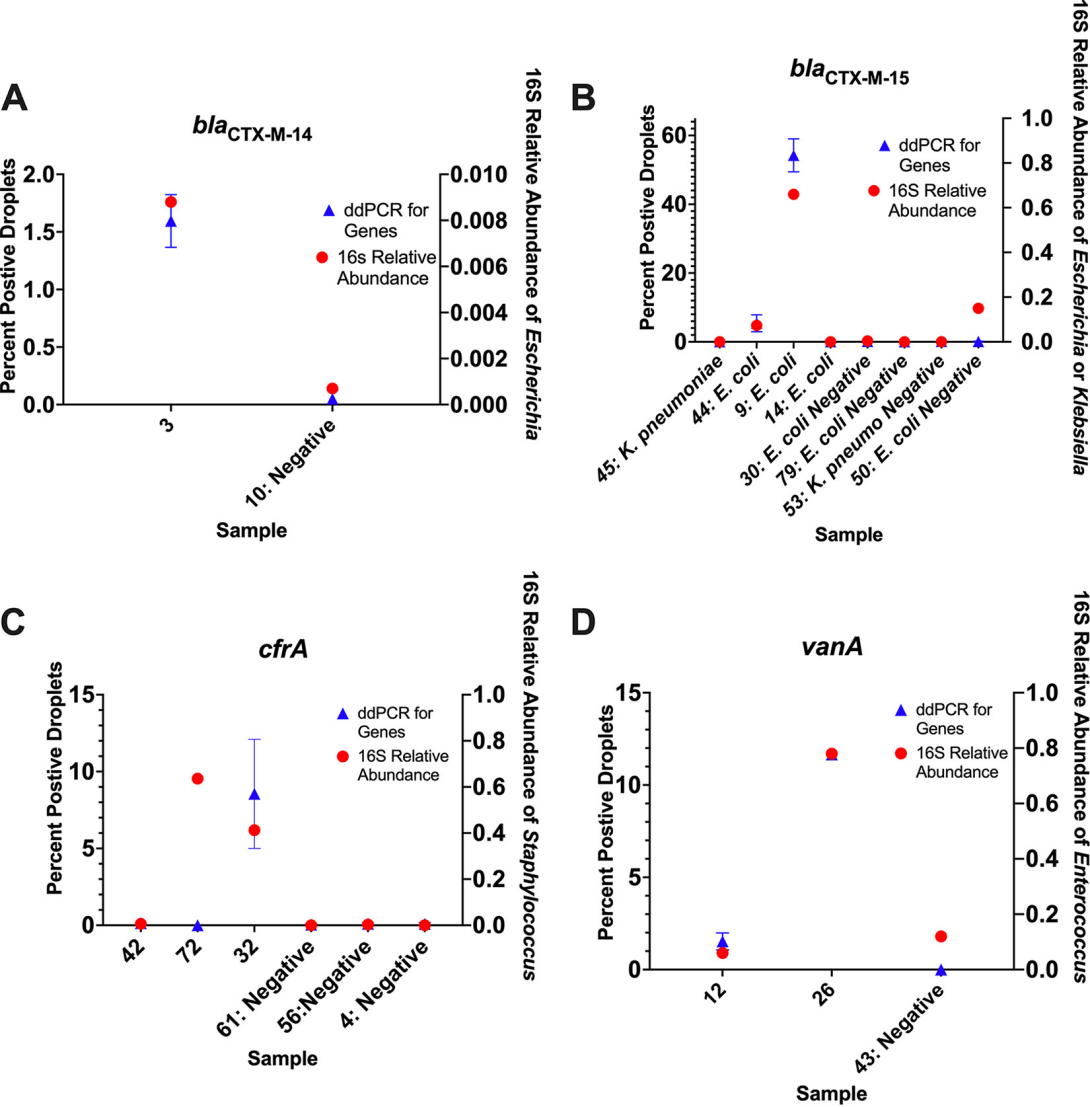
Presence of acquired resistance genes verified by ddPCR. This two-axis graphical depiction shows the 16S rRNA abundance of the genera of interest labeled on the right axis and percentage of positive droplets for the tested antibiotic resistance genes on the left axis. The *x* axis depicts each sample as the patient number followed by the pathogen being tested.

Given these results, we then tested individual stool samples via ddPCR for selected major resistance genes to include *bla*_CTX-M-14,_ *bla*_CTX-M-15,_ *cfrA*, and *vanA*. Stool samples from patients with K. pneumoniae infection were tested for *bla*_CTX-M-15_, and stool samples from patients with E. coli infection were tested for both *bla*_CTX-M-15_ and *bla*_CTX-M-14._ All stool samples from patients with S. epidermidis infection with available DNA were tested for *cfrA*, along with stool samples from patients with *Enterococcus* species infection for *vanA.* The one patient with an E. coli isolate positive for *bla*_CTX-M-14_ by WGS also had a corresponding stool sample positive by ddPCR (Fig. 5A). All 4 patients with BSI isolates that were positive for *bla*_CTX-M-15_ by WGS also had parallel stools positive via ddPCR (Fig. 5B). Two out of three patients with BSI isolates confirmed positive for *cfrA* by WGS had stool samples positive by ddPCR (Fig. 5C). For stool samples from patients with *Enterococcus* infection, *vanA* was detected by ddPCR in all of the stool samples with concurrent *vanA*-positive BSI isolates and zero droplet detection in the stool samples of patients infected by *vanA*-negative strains (Fig. 5D). We also determined that the percentage of positive droplets with antibiotic resistance gene detection mostly coincided with 16S abundance of the taxa from which they were derived. The only sample which was an outlier was among the *cfrA*-tested stools, in which there was a low percentage of positive droplets for *cfrA* but a relative high abundance of reads mapping the Staphylococcus. This is likely due to 16S amplification of another Staphylococcus species, not only S. epidermidis. These data show that antimicrobial resistance genes in pathogens causing serious infection in AML patients can be detected in stool samples.

## DISCUSSION

Bacteremia is a major cause of mortality among immunocompromised patients, and with the rising rates of antibiotic-resistant pathogens, it is increasingly imperative to optimize antimicrobial targeting (36, 37). In this cross-sectional observational study, we sought to determine if the abundance of specific taxa in either the oral and/or fecal microbiome could be correlated with the etiological agent of infection. Furthermore, we ascertained if the infectious species and antibiotic resistance determinants which were present in bacteremia isolates could be detected in the stool samples. Interestingly, we discovered that AML patients at the time of bacteremia rarely had >30% of 16S rRNA reads mapping to the genus of the infecting bacteria in either stool or oral samples, which previously has been used as the cutoff for intestinal domination, mainly in stem cell transplant patients. Moreover, we established that ddPCR of the stool was able to detect the suspected species causing infection as well as antibiotic resistance determinants genetically present within the infectious isolate. One of our key findings was that out of 63 patients who experienced bacteremia, only 7 had microbiome samples in which the infecting species reached the threshold of domination, i.e., >30% relative abundance. A number of studies show a correlation between microbiome domination and infection, but these studies predominantly focus on the gut microbiome, excluding the oral microbiome (6, 16, 21, 25, 38, 39). Previous findings in allo-HCT patients have shown that having intestinal domination was a major risk factor for bacteremia (15). However, even in the largest study examining this relationship, Stoma et al. recently reported domination events occurred in <50% of adult patients undergoing allo-HCT with Gram-negative BSIs (6). Although the timing of these events appears to be variable over transplant hospitalization, both BSIs and microbiota domination peaked at the same time, approximately 4 to 5 days posttransplantation. It has long been recognized that the intestines are the source of many bacteremias in immunocompromised or critically ill patients, and it would seem logical that higher levels of pathogenic bacteria would correlate with subsequent risk of translocation from the intestines into the bloodstream (13, 19, 22, 24, 40). However, there are many factors that likely limit 16S rRNA analysis of stool as a marker for bacteremia in immunocompromised patients, such as the imperfect relationship between stool and intestinally adherent bacteria, and alternative bacteremia sources such as upper GI, lung, and venous catheters. Finally, bacteremia, in theory, results from a single organism gaining entrance into the bloodstream, an unpredictable event which could occur even when pathogens are at low levels. Only 18 of our samples, between oral and stool, reached the threshold of >3% relative abundance of the same taxa which caused the infection. However, in all but 9 patients, the infectious genera were detectable in the stool or oral samples at the time of infection. Thus, together with previous findings, our data suggest that targeted qualitative assessment of mucosal pathogens and antimicrobial resistance determinants might be used in the future to optimize antimicrobial administration in immunocompromised patients with suspected nonfocal bacteremias.

Digital droplet PCR (ddPCR) was performed given that 16S rRNA sequencing has inherent limitations, including the fact that it produces relative instead of absolute results, as well as the fact that it cannot confirm our infectious agents down to a species level (41–43). We found that ddPCR was able to detect the suspected species causing infection in 66% of the stool samples tested, with *Enterococcus* spp. detected 100% of the time. When ddPCR was used to identify antibiotic resistance genes, there was little signal detected for known negative samples and consistent identification of positive samples across all four genes tested (Fig. 5). This serves to support that ddPCR could be a viable method for detecting key etiological agents of infection as well as important antibiotic resistance determinants in the stool at the time of bacteremia. Other studies have shown ddPCR has a lower detection limit, approximately one log better than quantitative PCR (qPCR) (44–46). This is particularly useful when testing small amounts of DNA in low-biomass samples, such as patients who are repeatedly treated with antibiotics (47). In addition to increased sensitivity, ddPCR provides the advantage of accelerated detection and superior discriminatory power among diverse subpopulations (48, 49). Patients need to be tested in a timely manner with the ability to detect continuous mutation and adaptation of microbes under selective pressure. Thus, ddPCR could be a valuable tool in improving treatment strategies well before culture identification and antibiogram results are available for blood samples.

Moreover, we found that some infections show enrichment of the infectious species in the oral cavity at the time of bloodstream infection, not just in the stool samples. Oral samples showed enrichment of Pseudomonas and Streptococcus at the time of BSI (Fig. 1). On the other hand, patients infected with E. coli showed enrichment of the genus Escherichia in stool samples compared to oral (Fig. 2) and significantly higher abundance in stool samples of patients with E. coli infection than those without (Fig. 1). While this also likely relates to the pathology of E. coli infections, we also saw enrichment of Pseudomonas in stool samples in patients with that infection compared to those with other etiological agents causing their BSI. These data indicate that oral sampling along with stool is likely needed to allow for microbiome monitoring to impact antimicrobial administration.

There were several limitations to this study. One is our lack of additional longitudinal microbiome samples given that the design of the study was to capture the microbiome at the time of BSI. Given the cross-sectional nature of the study, it is possible we could have missed a potential window to observe this occurrence in the GI tract. However, a cross-sectional study is more representative of the samples that would likely be collected clinically to determine potential risk for infection or modification of intervention strategies, as it is unlikely that patients would be followed longitudinally outside the research setting. Nevertheless, our analysis of our longitudinal cohort suggests that is unlikely dominance occurred beyond a small percentage of patients. Second, this study had no healthy control group or other infection group. So, although we could compare the results of patients with different etiological agents causing bacteremia, it was impossible for us to compare the composition of an uninfected patient’s microbiome or that of a non-BSI patient. With this in mind, additional studies specifically looking at bacteremia caused by each individual etiological agent would be beneficial. Last, results from 16S rRNA sequencing can vary greatly based on pipelines and programs used, the databases used for analysis, and the type of sequencing performed. So, although the differences we see in our 16S rRNA results and previous literature could be attributed to differences in sequencing and bioinformatic methodologies between studies, our results from the ddPCR confirm that our relative abundance values via 16S amplicon sequencing are likely adequate for purposes of this study.

These data show that the diverse array of pathogens causing bloodstream infection in AML patients is best detected utilizing both the oropharynx and stool when applying microbiome sampling. Additionally, we found that domination of the oral and gastrointestinal microbiome by the etiological agent at the time of BSI, or shortly thereafter, is not fundamental to developing a bloodstream infection in the AML setting. Moreover, these data provided the realization that 16S rRNA abundance thresholds of the stool or oral cavity are not consistent as a surveillance tool for bloodstream infections, thus impressing upon us the need for better, more consistent diagnostics and personalized medicine. As such, the addition of ddPCR of the stool for detection of life-threatening pathogens and antibiotic resistance determinants to the repertoire of patient monitoring should be considered, as it can identify patients for which to intervene in a more timely manner as well as improve empirical strategies to more personalized approaches.

## MATERIALS AND METHODS

### Study population and sample collection.

AML patients positive for a bacterial BSI were identified through collaboration with leukemia physicians and the clinical microbiology laboratory at the MD Anderson Cancer Center in Houston, Texas, from September 2014 through January 2019. The study protocol was approved by the MDACC Institutional Review Board (PA14-0641), and the study was conducted in compliance with the Declaration of Helsinki. Written informed consent was obtained from all participants before enrollment. To be included in the study, patients must have had a diagnosis of acute leukemia and a bacterial isolate from their bloodstream (two repeat positive blood cultures). Patients were approached for stool and oral sample collection at the time a blood culture was sent to the clinical microbiology lab for suspected infection. Thereafter, a follow-up for positive culture confirmation and collection of the bacterial isolate was completed with the clinical microbiology lab. We collected oral samples (buccal swabs) (*n* = 63), stool samples (*n* = 48), and bloodstream isolates (*n* = 64) from 63 patients (one patient had two unique positive blood cultures). Buccal swabs and stool samples were collected and stored as described previously (50). For oral samples, the range of collection time was from 6 days prior to confirmed positive culture up to the day of the culture. For stool samples, the range was from 5 days prior to confirmed positive culture to 7 days after. The BSI etiological agent (see Table S1 in the supplemental material) and antibiotic susceptibility profiles were collected from the clinical microbiology laboratory and electronic medical record, respectively.

### Whole-genome sequencing and analysis of bloodstream isolates.

DNA was isolated from bacteremia isolates using the MasterPure Gram-positive DNA purification kit (Lucigen). Whole-genome sequencing (WGS) was performed using Illumina NextSeq500 with 150-bp paired-end reads. The NGSQCToolkit 2.3.3 was used to filter high-quality reads, discarding an average of about 10% of reads. *De novo* assembly and gene prediction were completed using both SPAdes 3.7.1 and GeneMarkS 4.32. The assembly was further evaluated for quality by mapping read pairs and contig sequences using BWA 0.7.12.

Antimicrobial resistance gene protein sequences were made into a custom database as previously described, built via the merging of data from ARDB and CARD, including information on B-lactam alleles and/or mutations conferring B-lactam resistance (35). Alignments with an identity score of greater than 80%, mapped by USEARCH 8.1, were considered positive matches (51). The presence and absence of these sequences were compiled into a heatmap using the package pheatmap in R. *In silico* multilocus sequence typing (MLST) was performed using the Center for Genomic Epidemiology database (https://cge.food.dtu.dk/services/MLST/).

To more accurately identify the species of VGS isolates specifically, the genome sequences were assembled using SPAdes v3.15.5 in the BV-BRC Genome Assembly Service (https://www.bv-brc.org/) and were run through NCBI BLAST to determine closest related sequences. The *gyrB* gene sequences were then analyzed and compared to the list of amino acid residues previously found to differentiate between multiple VGS species (52).

### Specimen collection process and 16S rRNA sequencing.

The DNA from fecal samples was isolated by following the protocol from the QIAamp DNA stool minikit (Qiagen) to include a bead-beating step for lysis (53). For oral samples, briefly, buccal swabs were pretreated with lysozyme (10 mg/mL; Sigma) at 37°C for 1 h. After incubation, the mixture was treated with 20 μL proteinase K (10 mg/mL Qiagen) and 400 μL buffer ATL (Qiagen) at 56°C for 30 min. Subsequently, DNA was isolated using the QIAamp DNA minikit (catalog no. 51306; Qiagen) with a mechanical bead-beating lysis step (54). DNA extraction was performed from buccal swabs with a modified QIAamp kit (Qiagen) containing enzymatic and bead-beating lysis. In both scenarios, DNA was eluted in 50 μL of buffer AE and quantified using NanoDrop spectrophotometers (Fisher Scientific).

The V4 region of the 16S rRNA gene was amplified by PCR from 10 ng of extracted genomic DNA from the oral samples and 100 ng from stool samples using 515F and 806R primer pairs designed by Earth Microbiome Project (55). The amplicon libraries were sequenced using the Illumina MiSeq platform with a 2 × 250-bp paired-end protocol. The reads were demultiplexed via QIIME and then merged and dereplicated for chimeras utilizing VSEARCH. Following denoising and chimera calling using the UNOISE3 command (56), unique sequences were taxonomically classified with mothur using the SILVA database version 138. An operational taxonomic unit (OTU) table was then generated by using USEARCH, and alpha and beta diversity metrics were determined in QIIME. Sample sequences were rarefied to the number of the sample with the least sequences (1792) (41). Utilizing the oral and stool samples collected from our cohort, we determined the correlation between the etiological agent and the taxa present (>0% reads mapping to the infectious genera), colonizing (>3% reads mapping to the infectious genera), or dominating (>30% mapping to the infectious genera) the microbiome at oral and stool sites (Table 1).

### Confirmation of species-level specificity and detection of antibiotic resistance genes by digital droplet PCR.

In order to confirm bacterial abundances at the species level, digital droplet PCR was used to analyze the load of each infectious agent in stool samples utilizing the Bio-Rad QX200 droplet digital PCR system. Genomic DNA was digested with the HF HindIII enzyme (NEB) for 1 h at 37°C and then heat inactivated at 80°C for 20 min. Following digestion, samples were diluted to either 10 ng/μL or 1 ng/μL. Samples were run in duplicate with each experiment performed in duplicate on a different day. Stool samples with no reads mapping to the same genera were used at controls. Primers, probes, and PCR conditions for species-specific ddPCR are located in Table S2. The same protocol was used to ascertain if antibiotic resistance genes could be found in patient stool samples, namely, *bla*_CTX-M-15_, *bla*_CTX-M-14_, *cfrA*, and *vanA*. When BSI isolates were identified to have any of the above-described resistance genes via WGS, we performed ddPCR on the stool samples collected from patients infected by those isolates. We also tested stool samples from patients who had BSIs with the same species but whose bacteremia isolates did not have those resistance genes seen via WGS as controls. Primers, probes, and PCR conditions for antibiotic resistance gene detection in the stool can be obtained from Table S3.

### Statistical analysis.

Pairwise testing, as well as bivariate comparison and plotting, were performed in GraphPad Prism 6 (GraphPad Software) using the Mann-Whitney U test and Fisher’s exact tests. Linear discriminant analysis for effect size (LEfSe) was performed via the Galaxy portal to compare taxonomic abundances between groups (57, 58).

### Data availability.

The 16S rRNA sequences from human oral and stool samples and whole-genome sequences from infection isolates were deposited in the NCBI Sequence Read Archive (http://www.ncbi.nlm.nih.gov/sra) under the BioProject accession numbers PRJNA913942 and PRJNA913921.
