# Supplementary material for: Contribution of the Oral and Gastrointestinal Microbiomes to Bloodstream Infections in Leukemia Patients
Source: Microbiol Spectr. 2023 Apr 6;11(3):e00415-23. doi: 10.1128/spectrum.00415-23 (PMC10269818; doi:10.1128/spectrum.00415-23)
Supplement: Supplemental file 1 — Tables S1 to S3 and Fig. S1 to S7. Download spectrum.00415-23-s0001.pdf, PDF file, 1.1 MB [file spectrum.00415-23-s0001.pdf]

Supplemental Table 1. Phenotypic antibiotic resistances of infectious isolates

Patient	Infectious Organism	AMK	ATM	FEP	CAZ	CIP	CST	IPM	MEM	TZP	TIM	TOB	CRO	MXF	PEN	TET	TGC	VAN	AMC	ETP	SXT	CLI	ERY	GEN	LVX	LZD	NIT	OXA	RIF	AMP	SAM	CTX	CPD	CEF	DAP	Q-D	STR	CZA	MIN	C/T	CPT
B001	*Pseudomonas aeruginosa*	S	S	I	S	R	S	R	R	S	R	S	-	-	-	-	-	-	-	-	-	-	-	-	-	-	-	-	-	-	-	-	-	-	-	-	-	-	-	-	-
B002	*Streptococcus mitis*	-	-	-	-	-	-	-	-	-	-	-	S	R	S	R	S	S	-	-	-	-	-	-	-	-	-	-	-	-	-	-	-	-	-	-	-	-	-	-	-
B003	*Escherichia coli*	S	-	S	-	R	-	S	S	S	I	S	-	R	-	-	S	-	I	S	R	-	-	-	-	-	-	-	-	-	-	-	-	-	-	-	-	-	-	-	-
B004	*Staphylococcus epidermidis*	-	-	-	-	-	-	-	-	-	-	-	-	-	-	R	S	S	-	-	-	S	R	S	R	S	S	R	I	-	-	-	-	-	-	-	-	-	-	-	-
B005	*Streptococcus oralis*	-	-	-	-	-	-	-	-	-	-	-	S	R	I	R	S	S	-	-	-	-	-	-	-	-	-	-	-	-	-	-	-	-	-	-	-	-	-	-	-
B006	*Staphylococcus epidermidis*	-	-	-	-	-	-	-	-	-	-	-	-	-	-	R	S	S	-	-	-	R	R	R	R	-	S	R	S	-	-	-	-	-	-	-	-	-	-	-	-
B007	*Pseudomonas aeruginosa*	S	S	S	-	S	-	S	-	S	S	S	-	-	-	-	-	-	-	-	-	-	-	-	-	-	-	-	-	-	-	-	-	-	-	-	-	-	-	-	-
B008	*Streptococcus mitis*	-	-	-	-	-	-	-	-	-	-	-	S	R	I	R	S	S	-	-	-	-	-	-	-	-	-	-	-	-	-	-	-	-	-	-	-	-	-	-	-
B009	*Escherichia coli*	S	-	S	-	R	-	S	-	S	R	R	-	R	-	-	S	-	R	S	R	-	-	-	-	-	-	-	-	-	-	-	-	-	-	-	-	-	-	-	-
B010	*Escherichia coli*	S	S	S	S	R	-	S	S	S	-	S	S	R	-	-	S	-	-	S	S	-	-	S	R	-	S	-	-	R	R	S	S	R	-	-	-	-	-	-	-
B011	*Staphylococcus aureus (MRSA)*	-	-	-	-	-	-	-	-	-	-	-	-	-	-	S	S	S	-	-	S	S	R	S	R	S	S	R	S	-	-	-	-	-	S	-	-	-	-	-	-
B012	*Enterococcus faecium, VRE*	-	-	-	-	-	-	-	-	-	-	-	-	-	-	R	-	R	-	-	-	-	-	R	R	I	S	-	-	R	-	-	-	-	-	S	S	-	-	-	-
B013	*Streptococcus oralis*	-	-	-	-	-	-	-	-	-	-	-	R	S	R	R	S	S	-	-	-	-	-	-	-	S	-	-	-	-	-	-	-	-	S	-	-	-	-	-	-
B014	*Pseudomonas aeruginosa*	S	S	S	S	S	-	S	S	S	S	S	-	-	-	-	-	-	-	-	-	-	-	-	-	-	-	-	-	-	-	-	-	-	-	-	-	-	-	-	-
B015	*Staphylococcus epidermidis*	-	-	-	-	-	-	-	-	-	-	-	-	-	-	S	S	S	-	-	-	R	R	R	R	R	S	R	S	-	-	-	-	-	-	-	-	-	-	-	-
B016	*Streptococcus sanguinis*	-	-	-	-	-	-	-	-	-	-	-	S	R	S	R	S	S	-	-	-	-	-	-	-	-	-	-	-	-	-	-	-	-	-	-	-	-	-	-	-
B017	*Staphylococcus epidermidis*	-	-	-	-	-	-	-	-	-	-	-	-	-	-	S	S	S	-	-	-	R	R	R	R	-	S	R	S	-	-	-	-	-	-	-	-	-	-	-	-
B018	*Enterococcus faecalis*	-	-	-	-	-	-	-	-	-	-	-	-	-	-	R	-	S	-	-	-	-	-	R	R	S	S	-	-	S	-	-	-	-	S	R	-	-	-	-	-
B019	*Escherichia coli*	S	R	I	R	R	-	S	S	S	-	S	R	R	-	-	S	-	-	S	R	-	-	S	R	-	I	-	-	R	R	R	R	R	-	-	-	-	-	-	-
B021	*Streptococcus mitis*	-	-	-	-	-	-	-	-	-	-	-	S	S	R	S	S	S	-	-	-	-	-	-	-	-	-	-	-	-	-	-	-	-	-	-	-	-	-	-	-
B022	*Pseudomonas aeruginosa*	S	S	S	S	S	-	S	S	S	I	S	-	-	-	-	-	-	-	-	-	-	-	-	-	-	-	-	-	-	-	-	-	-	-	-	-	-	-	-	-
B023	*Escherichia coli*	S	S	S	S	R	-	S	S	S	-	S	S	R	-	-	S	-	-	S	R	-	-	R	R	-	S	-	-	R	I	S	-	R	-	-	-	-	-	-	-
B024	*Streptococcus mitis*	-	-	-	-	-	-	-	-	-	-	-	S	R	S	S	S	S	-	-	-	-	-	-	-	-	-	-	-	-	-	-	-	-	-	-	-	-	-	-	-
B025	*Pseudomonas aeruginosa*	R	I	R	S	R	S	R	R	R	-	R	-	-	-	-	-	-	-	-	-	-	-	-	-	-	-	-	-	-	-	-	-	-	-	-	-	-	-	-	-
B026	*Enterococcus faecium*	-	-	-	-	-	-	-	-	-	-	-	-	-	-	R	-	R	-	-	-	-	-	R	R	R	-	-	-	R	-	-	-	-	S	R	-	-	-	-	-
B027	*Staphylococcus aureus (MRSA)*	-	-	-	-	-	-	-	-	-	-	-	-	-	-	S	S	S	-	-	S	R	T	S	R	S	-	R	S	-	-	-	-	-	S	-	-	-	-	-	-
B028	*Streptococcus mitis*	-	-	-	-	-	-	-	-	-	-	-	S	S	S	R	S	S	-	-	-	-	-	-	-	-	-	-	-	-	-	-	-	-	-	-	-	-	-	-	-
B029	*Escherichia coli*	S	S	S	S	R	-	S	S	S	-	S	S	R	-	-	S	-	-	S	S	-	-	S	R	-	-	-	-	R	I	S	R	R	-	-	-	-	-	-	-
B030	*Escherichia coli*	S	S	S	S	R	-	-	S	S	-	S	S	-	-	-	S	-	-	S	R	-	-	S	R	-	S	-	-	R	I	S	S	I	-	-	-	-	-	-	-
B032	*Staphylococcus epidermids*	-	-	-	-	-	-	-	-	-	-	-	-	-	-	R	S	S	-	-	-	R	R	R	R	-	-	R	S	-	-	-	-	-	-	-	-	-	-	-	-
B034	*Staphylococcus epidermids*	-	-	-	-	-	-	-	-	-	-	-	-	-	-	R	S	S	-	-	-	S	R	S	R	S	-	R	I	-	-	-	-	-	-	-	-	-	-	-	-
B035	*Enterococcus faecalis*	-	-	-	-	-	-	-	-	-	-	-	-	-	-	R	-	S	-	-	-	-	-	-	-	S	-	-	-	S	-	-	-	-	S	-	-	-	-	-	-
B036	*Pseudomonas aeruginosa*	S	I	S	S	S	-	S	S	S	-	S	-	-	-	-	-	-	-	-	-	-	-	-	-	-	-	-	-	-	-	-	-	-	-	-	-	-	-	-	-
B037B	*Streptoccoccus salivarius*	-	-	-	-	-	-	-	-	-	-	-	S	S	I	S	S	S	-	-	-	-	-	-	-	-	-	-	-	-	-	-	-	-	-	-	-	-	-	-	-
B038	*Streptococcus parasanguinis*	-	-	-	-	-	-	-	-	-	-	-	S	I	I	R	S	S	-	-	-	-	-	-	-	-	-	-	-	-	-	-	-	-	-	-	-	-	-	-	-
B039	*Streptococcus mitis*	-	-	-	-	-	-	-	-	-	-	-	S	R	I	R	-	S	-	-	-	-	-	-	-	-	-	-	-	-	-	-	-	-	-	-	-	-	-	-	-
B041	*Escherichia coli*	S	R	S	R	R	-	S	S	S	-	I	R	R	-	-	-	-	S	S	R	-	-	R	R	-	-	-	-	R	S	R	R	-	-	-	-	-	-	-	-
B042	*Staphylococcus epidermidis*	-	-	-	-	-	-	-	-	-	-	-	-	-	-	-	-	S	-	-	-	-	-	-	-	R	-	R	-	-	-	-	-	-	S	-	-	-	-	-	-
B043	*Enterococcus faecium, VRE*	-	-	-	-	-	-	-	-	-	-	-	-	-	-	-	-	R	-	-	-	-	-	-	-	S	-	-	-	S	-	-	-	-	S	-	-	-	-	-	-
B044	*Escherichia coli*	S	R	R	R	R	-	-	S	S	-	R	R	-	-	-	-	-	-	S	-	-	-	R	-	-	-	-	-	-	R	-	-	-	-	-	-	-	-	-	-
B045	*Klebsiella pneumoniae*	S	R	R	R	R	-	S	R	R	-	R	R	-	-	-	R	-	-	R	-	-	-	-	R	-	-	-	-	-	R	-	-	-	-	-	-	S	R	-	-
B047	*Pseudmonas aeruginosa*	I	-	I	S	R	-	-	S	I	-	S	-	-	-	-	-	-	-	-	-	-	-	R	-	-	-	-	-	-	-	-	-	-	-	-	-	-	-	-	-
B048	*Escherichia coli*	S	S	S	S	R	-	-	S	S	-	S	S	-	-	-	-	-	-	S	-	-	-	S	-	-	-	-	-	-	R	-	-	-	-	-	-	-	-	-	-
B049	*Staphylococcus aureus*	-	-	-	-	-	-	-	-	-	-	-	-	-	-	-	-	S	-	-	S	-	-	-	-	S	-	S	-	-	-	-	-	-	S	-	-	-	-	-	-
B050	*Escherichia coli*	S	S	R	R	R	-	-	S	R	-	R	R	-	-	-	-	-	-	S	-	-	-	R	-	-	-	-	-	-	R	-	-	-	-	-	-	-	-	-	-
B052	*Staphylococcus aureus*	-	-	-	-	-	-	-	-	-	-	-	-	-	-	-	-	S	-	-	S	-	-	-	-	S	-	S	-	-	-	-	-	-	S	-	-	-	-	-	-
B053	*Klebsiella pneumonaie*	S	S	S	-	S	-	-	S	S	-	S	S	-	-	-	-	-	-	S	-	-	-	S	-	-	-	-	-	-	S	S	-	-	-	-	-	-	-	-	-
B054	*Enterococcus faecalis*	-	-	-	-	-	-	-	-	-	-	-	-	-	-	-	-	S	-	-	-	-	-	-	-	-	-	-	-	S	-	-	-	-	S	-	-	-	-	-	-
B055	*Escherichia coli*	S	S	S	S	S	-	S	S	S	-	S	S	R	-	-	-	-	I	S	R	-	-	S	R	-	-	-	-	R	R	S	-	-	-	-	-	-	-	-	-
B056	*Staphylocccus epidermidis*	-	-	-	-	-	-	-	-	-	-	-	-	-	-	-	-	S	-	-	-	-	-	-	-	R	-	R	-	-	-	-	-	-	S	-	-	-	-	-	-
B059	*Streptococcus oralis*	-	-	R	-	-	-	-	-	-	-	-	-	-	I	-	-	S	-	-	-	-	-	-	R	-	-	-	-	-	-	S	-	-	S	-	-	-	-	-	-
B061	*Staphylococcus epidermidis*	-	-	-	-	-	-	-	-	-	-	-	-	-	-	-	-	S	-	-	-	-	-	-	-	S	-	R	-	-	-	-	-	-	S	-	-	-	-	-	-
B065	*Staphylococcus epidermidis*	-	-	-	-	-	-	-	-	-	-	-	-	-	-	-	-	S	-	-	-	-	-	-	-	S	-	R	-	-	-	-	-	-	S	-	-	-	-	-	-
B066	*Pseudomonas aeruginosa*	S	-	I	R	S	-	-	R	R	-	S	-	-	-	-	-	-	-	-	-	-	-	S	-	-	-	-	-	-	-	-	-	-	-	-	-	-	-	S	-
B067	*Klebsiella pneumoniae*	S	S	S	S	S	-	-	S	S	-	S	S	-	-	-	-	-	-	S	-	-	-	S	-	-	-	-	-	S	-	-	-	-	-	-	-	-	-	-	-
B068	*Pseudomonas aeruginosa*	S	-	I	S	S	-	S	S	S	-	S	-	-	-	-	-	-	-	-	-	-	-	-	S	-	-	-	-	-	-	-	-	-	-	-	-	-	-	-	-
B069	*Staphylococcus epidermidis (B069)*	-	-	-	-	-	-	-	-	-	-	-	-	-	-	-	-	S	-	-	-	-	-	-	-	R	-	R	-	-	-	-	-	-	S	-	-	-	-	-	-
B069A	*Streptococcus parasanguinis (B069A)*	-	-	R	-	-	-	-	-	-	-	-	R	-	R	-	-	S	-	-	-	-	-	-	R	-	-	-	-	-	-	-	-	-	-	-	-	-	-	-	-
B070	*Streptococcus oralis*	-	-	S	-	-	-	-	-	-	-	-	S	-	I	-	-	S	-	-	-	-	-	-	R	-	-	-	-	-	-	-	-	-	-	-	-	-	-	-	-
B072	*Staphylococcus epidermidis*	-	-	-	-	-	-	-	-	-	-	-	-	-	-	R	-	S	-	-	-	-	-	-	-	R	-	R	-	-	-	-	-	-	S	-	-	-	R	-	S
B075	*Pseudomonas aeruginosa*	S	-	R	R	S	-	-	R	I	-	S	-	-	-	-	-	-	-	-	-	-	-	S	-	-	-	-	-	-	-	-	-	-	-	-	-	-	-	S	-
B077	*Klebsiella pneumoniae*	S	R	R	R	R	-	-	S	I	-	-	-	-	-	-	-	-	-	S	-	-	-	R	-	-	-	-	-	-	R	-	-	-	-	-	-	S	-	-	-
B078	*Methicillin resistant Staphylococcus aureus*	-	-	-	-	-	-	-	-	-	-	-	-	-	-	-	-	S	-	-	S	-	-	-	-	S	-	R	-	-	-	-	-	-	S	-	-	-	-	-	-
B079	*Escherichia coli*	S	S	S	I	R	-	-	S	S	-	S	S	-	-	-	-	-	-	S	-	-	-	S	-	-	-	-	-	-	R	-	-	-	-	-	-	-	-	-	-

R for resistant, I for intermediate, and S for susceptible. Antibiotic abbreviations follow AAC abbreviation standards.

Supplemental Table 2: ddPCR Primers and Probes to Confirm of Infectious Agents in Patient Stool Samples.

Enterococcus spp.	Forward	GAGAAATTCCAAACGAACTTG	Tm: 50ºC
	Reverse	CAGTGCTCTACCTCCATCATT	Tm: 54.2ºC
	Probe, Dye and Quencher	TGGTTCTCTCCGAAATAGCTTTAGGGCTA<br>6-FAM; BHQ-1	Tm: 60.3ºC
Escherichia coli	Forward	TCACGCCGTATGTTATTG	Tm: 49.4ºC
	Reverse	GTCGGTAATCACCATTCC	Tm: 49.8ºC
	Probe, Dye and Quencher	TGCCAGTTCAGTTCGTTGTTCAC<br>6-FAM; BHQ-1	Tm: 58ºC
Klebsiella pneumoniae	Forward	GGGCGAGGTTTACGTCTCAA	Tm: 57.8ºC
	Reverse	GCGTGTGGATAAGAGGTGCG	Tm: 58.2ºC
	Probe, Dye and Quencher	CCACCACGAGCGGCTGCC<br>6-FAM; BHQ-1	Tm: 64.7ºC
Pseudomonas aeruginosa	Forward	TTCAACGAGAACCTGCTCTG	Tm: 55.5ºC
	Reverse	CGGCCTCGATGTAGTTGTT	Tm: 54.9ºC
	Probe, Dye and Quencher	CTTCACCAACAACATCCCGCAGC<br>6-FAM; BHQ-1	Tm: 59ºC
Staphylococcus aureus	Forward	GAGTTTGGTGCCTTTACAGATAG	Tm: 53.4ºC
	Reverse	AGCAAGCTTTAACTCATAGTGG	Tm: 52.8ºC
	Probe, Dye and Quencher	TGCCATACAGTCATTTCACGCAAACTG<br>6-FAM; BHQ-1	Tm: 59.8ºC
	Forward	ATCAAAAAGTTGGCGAACCTTTTCA	Tm: 56.9ºC
	Reverse	CAAAAGAGCGTGGAGAAAAGTATC	Tm: 54.1ºC

Staphylococcus epidermidis	Probe, Dye and Quencher	CCATTTGCATAGTCTGATTGCTCAAAGTCT Tm: 59.0ºC<br>6-FAM; BHQ-1
Streptococcus mitis	Forward	TGCCCTTTCCACTCAATTAGAC Tm: 54.9ºC
	Reverse	GAACCGTTGTTCCAGTCCTATC Tm: 55.3ºC
	Probe, Dye and Quencher	TACCGTCGTGGTCATGTTGTTGCT Tm: 56.2ºC<br>6-FAM; BHQ-1
Streptococcus oralis	Forward	GATTACCTTTGCGGAACGTTTG Tm: 54.8ºC
	Reverse	GGCTAGAAGAGAGAGGAGTTGA Tm: 55.3ºC
	Probe, Dye and Quencher	CGATCAATCTGGTGCCCTTCGTGA Tm: 61.1ºC<br>6-FAM; BHQ-1

Supplemental Table 3: ddPCR Primers and Probes to Confirm Antibiotic Resistance Gene Presence in Patient Stool Samples.

CTX-M-15	Forward	GACTGGGTGTGGCATTGATTAAC	Tm: 64°C
	Reverse	CAGATTCGGTTCGCTTTCACTTT	Tm: 64°C
	Probe, Dye and Quencher	ATGAGCGCTTTGCGATGTGCAGCA<br>6-FAM; BHQ-1	Tm: 71°C
CTX-M-14	Forward	TGACGTGGCTCAAAGGCAATA	Tm: 64°C
	Reverse	TATCATTGGTGGTGCCGTAGTC	Tm: 64°C
	Probe, Dye and Quencher	TGGACTGTGGGTGATAAGACCGGCA<br>6-FAM; BHQ-1	Tm: 71°C
CfrA	Forward	GATTATATCATGTTGCCTGGTGTAAATG	Tm: 63°C
	Reverse	CCATACATCTCAGGTGCACTTATTG	Tm: 63°C
	Probe, Dye and Quencher	TCTTGAGCATGCAAACGAGTTGTTAGCCT<br>6-FAM; BHQ-1	Tm: 70°C
VanA	Forward	TCCTGACCAGTCTACGACCAATAC	Tm: 65°C
	Reverse	CGGTAATCAAGCAGACTATGCGATAC	Tm: 65°C
	Probe, Dye and Quencher	ACAATCCGGGTGTACTTGTTTCCTTTGCA<br>6-FAM; BHQ-1	Tm: 70°C

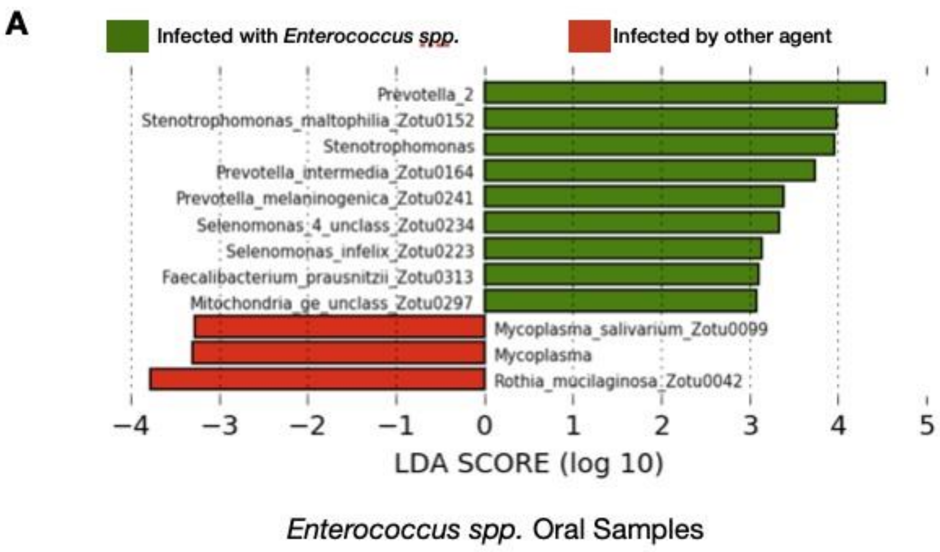

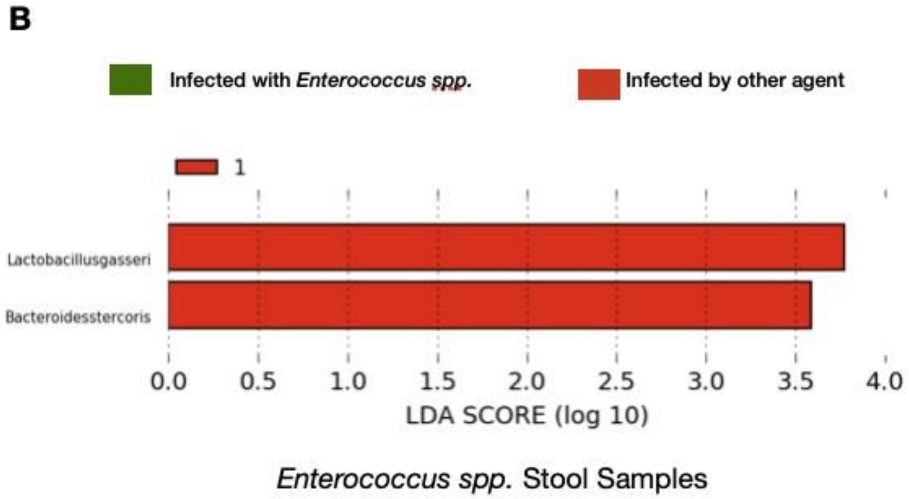

Supplemental Figure 1. LEfSe analysis of *Enterococcus spp.* infections. Linear discriminant analysis of effect size was done on oral (A) and stool (B) samples to compare the microbiomes of patients with an *Enterococcus spp.* infection to those without. Organisms with a red bar show enrichment in patients without an *Enterococcus spp.* infection while those with a green bar show enrichment in patients with an *Enterococcus spp.* infection.

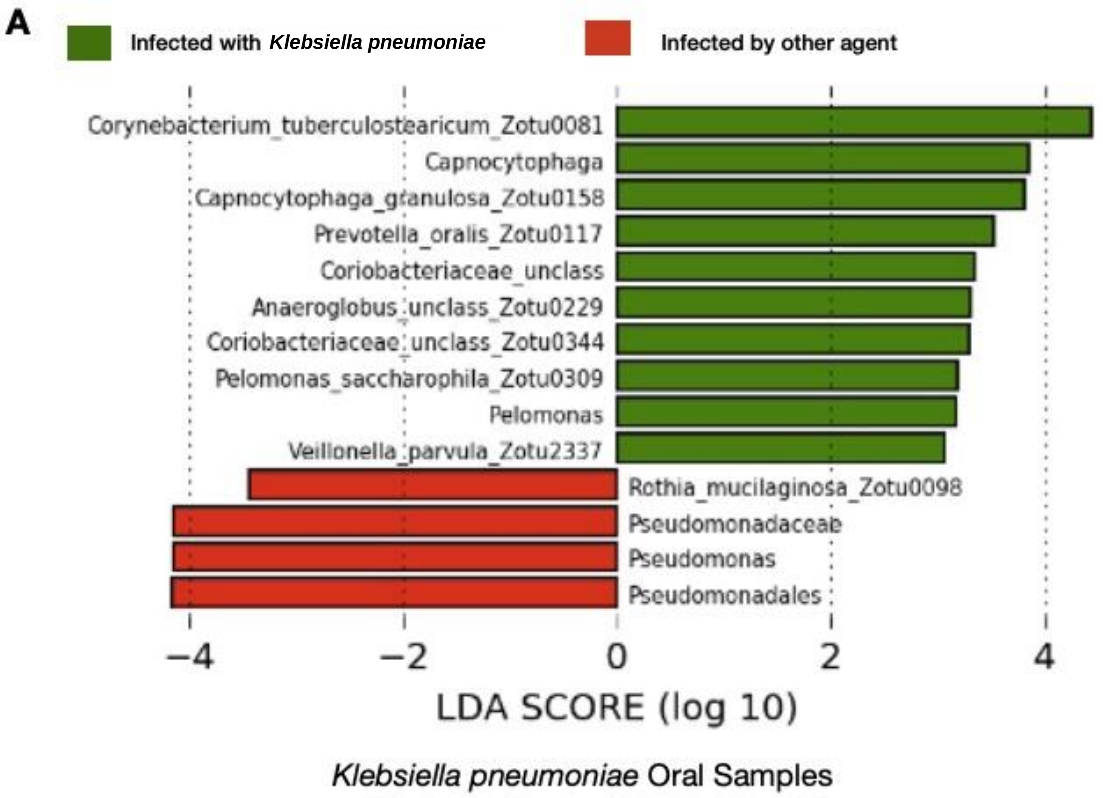

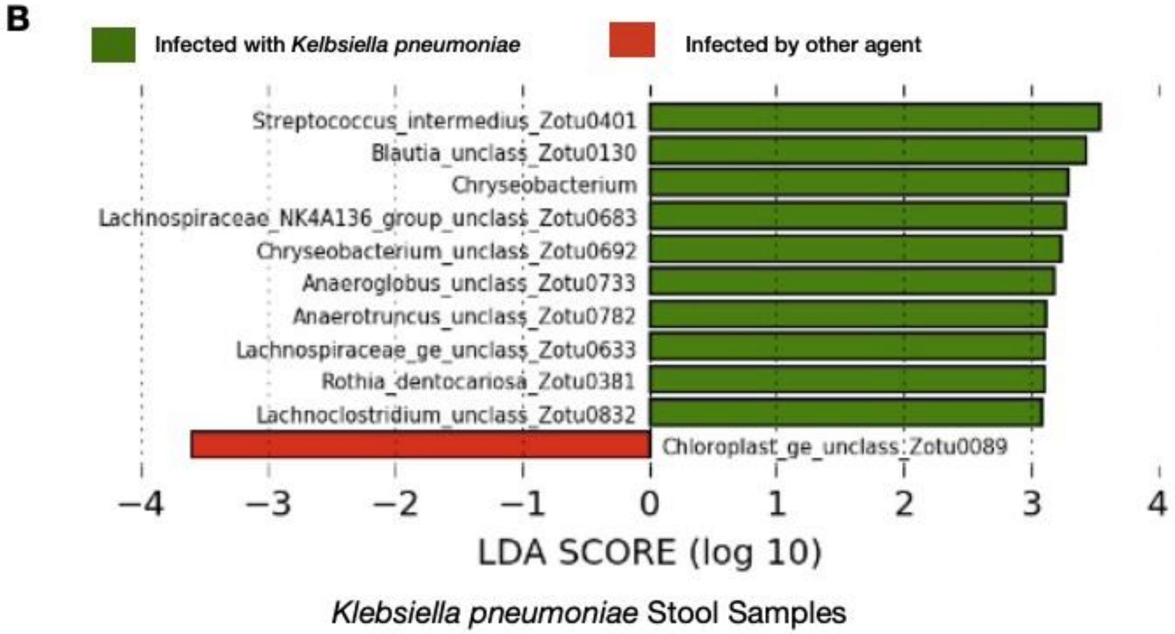

Supplemental Figure 2. LEfSe analysis of *Klebsiella pneumoniae* infections. Linear discriminant analysis of effect size was done on oral (A) and stool (B) samples to compare the microbiomes of patients with a *Klebsiella pneumoniae* infection to those without. Organisms with a red bar show enrichment in patients without a *K. pneumoniae* infection while those with a green bar show enrichment in patients with a *K. pneumoniae* infection.

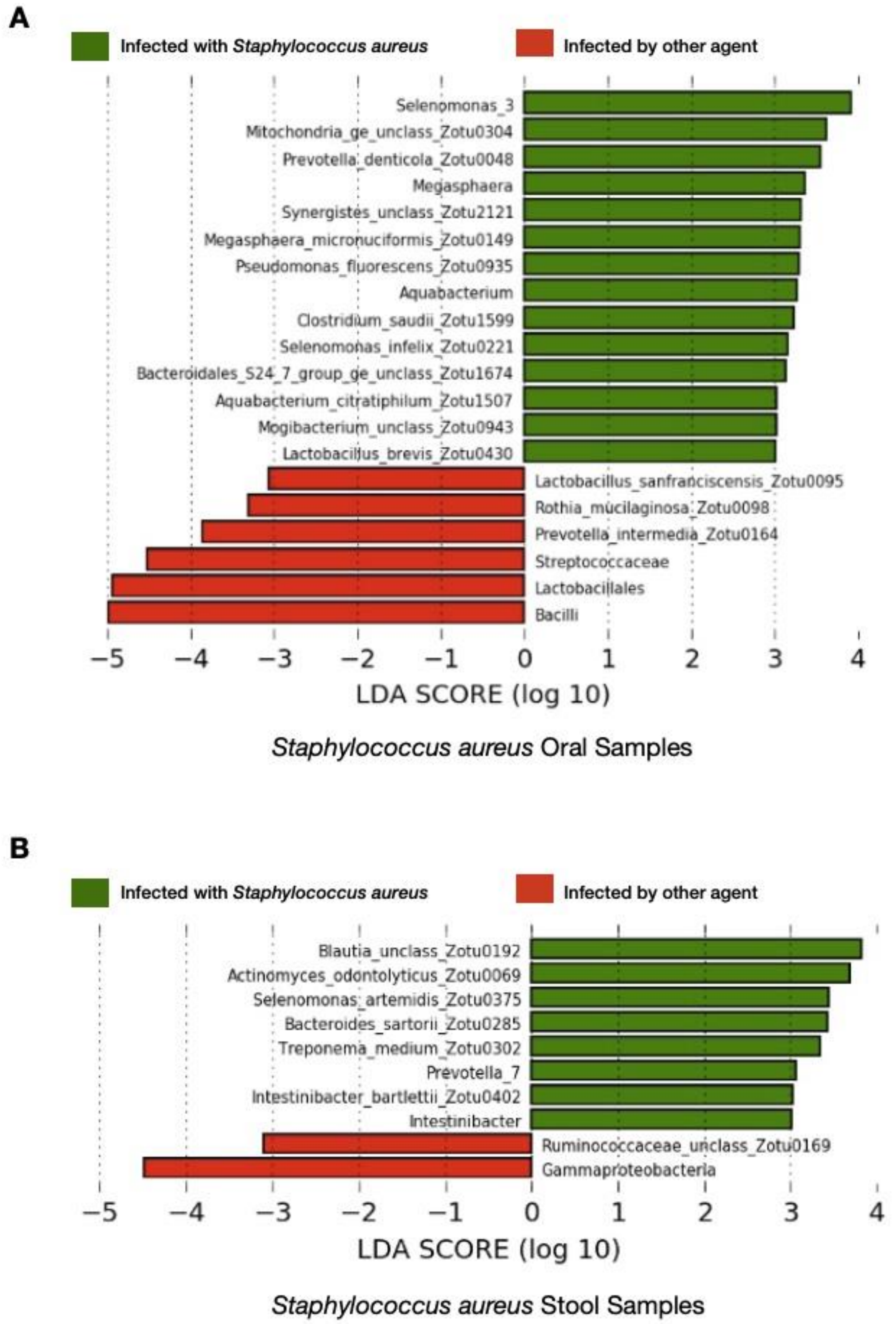

Supplemental Figure 3. LEfSe analysis of *Staphylococcus aureus* infections. Linear discriminant analysis of effect size was done on oral (A) and stool (B) samples to compare the microbiomes of patients with a *Staphylococcus aureus* infection to those without. Organisms with a red bar show enrichment in patients without a *S. aureus* infection while those with a green bar show enrichment in patients with a *S. aureus* infection.

A

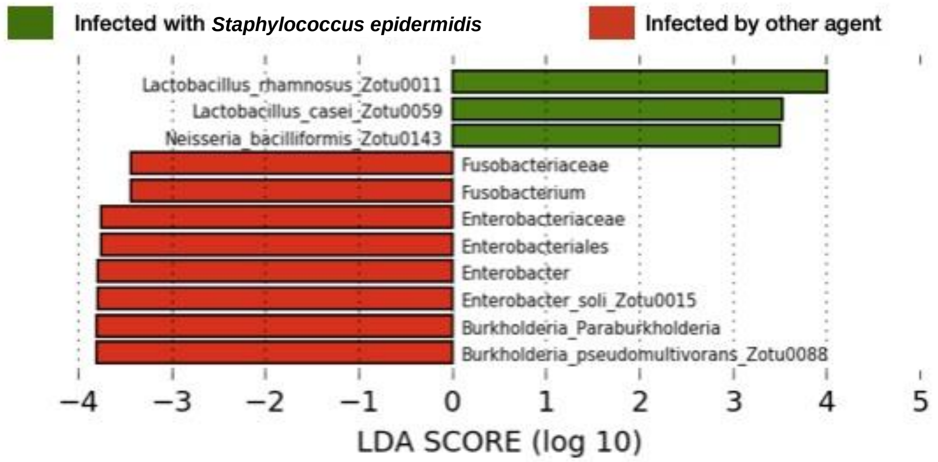

Staphylococcus epidermis Oral Samples

B

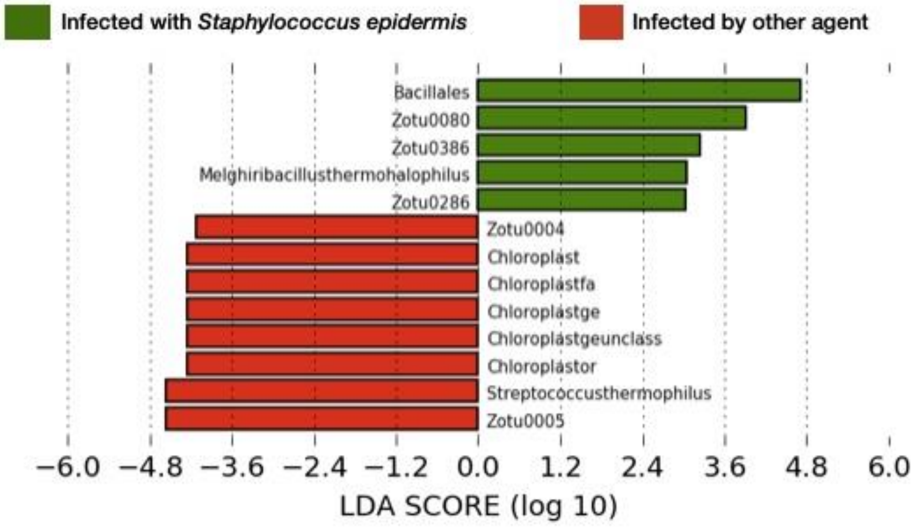

Staphylococcus epidermis Stool Samples

Supplemental Figure 4. LEfSe analysis of *Staphylococcus epidermidis* infections. Linear discriminant analysis of effect size was done on oral (A) and stool (B) samples to compare the microbiomes of patients with a *Staphylococcus epidermidis* infection to those without. Organisms with a red bar show enrichment in patients without a *S. epidermidis* infection while those with a green bar show enrichment in patients with a *S. epidermidis* infection.

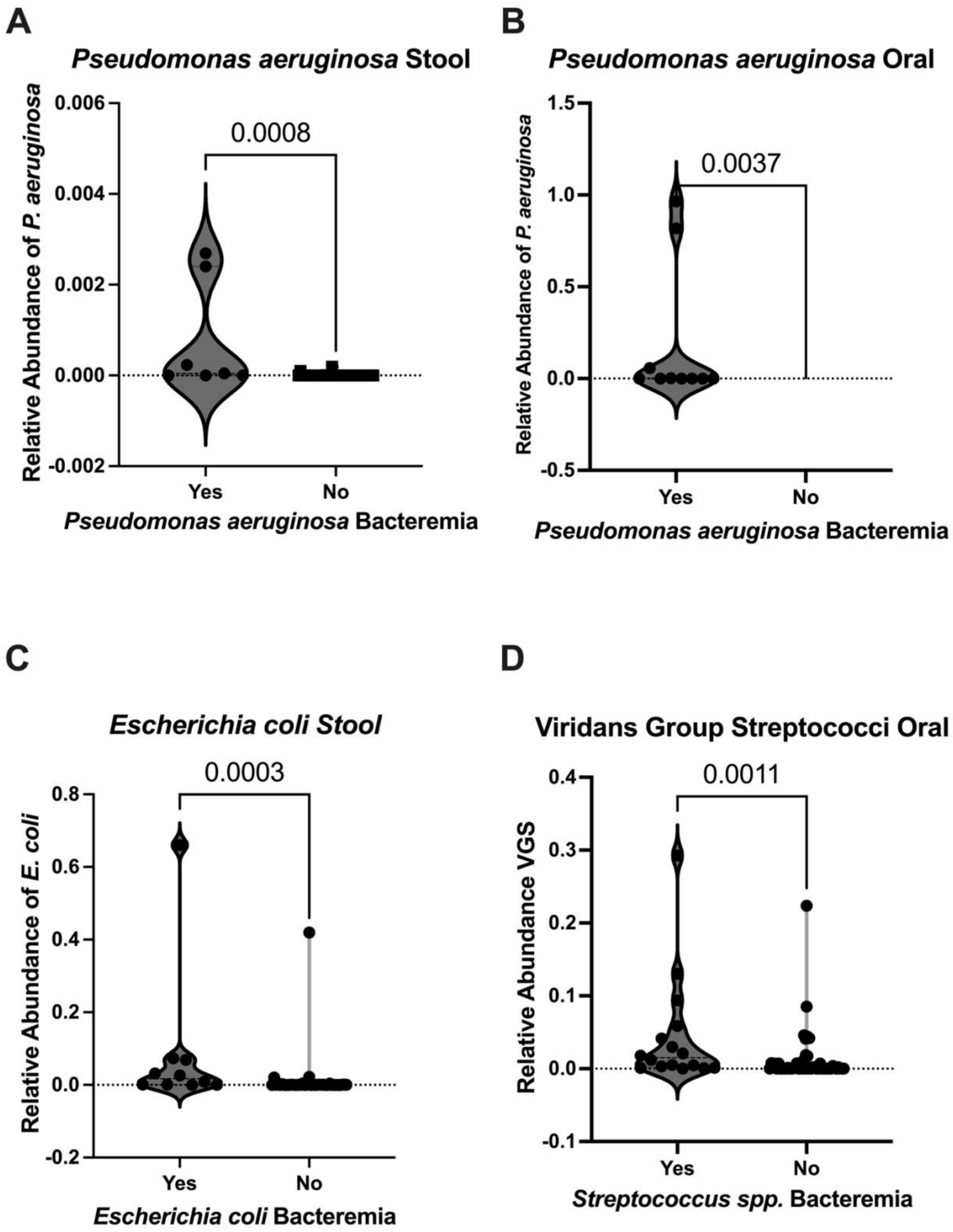

Supplemental Figure 5. Distribution of infectious taxa between patients with and without respective bacteremias. Mann-Whitney testing demonstrates that patients with *E. coli* and *P. aeruginosa* bacteremia had higher abundance of the infectious taxa in their stool. Additionally, patients with *P. aeruginosa* and VGS bacteremia had increased abundance of the infectious taxa in the oral samples.

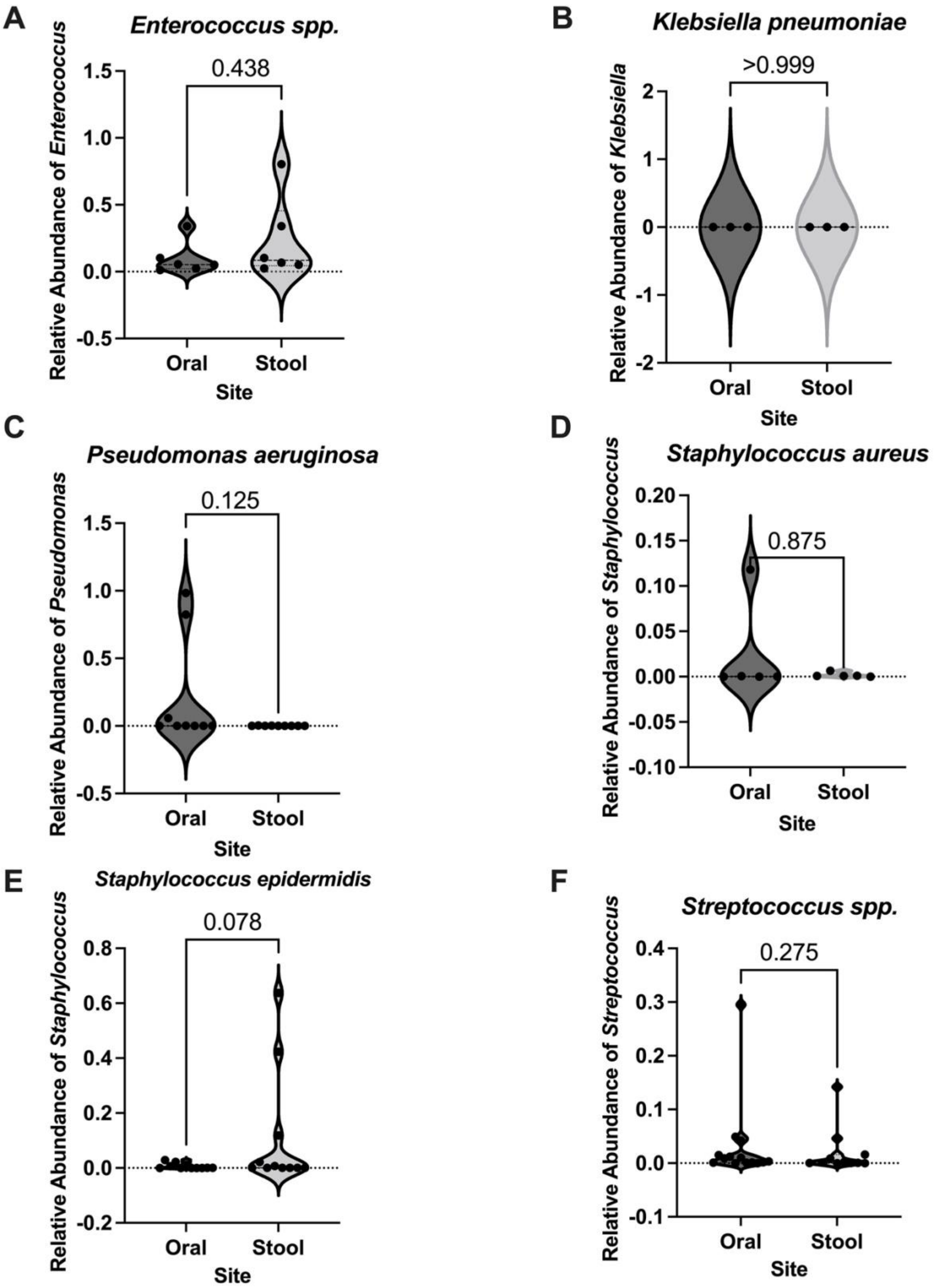

Supplemental Figure 6. Demonstration of differences in abundance based on site and species specificity. Demonstration of differences in abundance based on site specificity per species. Mann-Whitney testing was performed using the relative abundance of each genera compared between stool and oral samples of patients infected with A) *Enterococcus spp.*, B) *Klebsiella pneumoniae,* C) *Pseudomonas aeruginosa*, D) *Staphylococcus aureus*, E) *Staphylococcus epidermidis* and F) *Streptococcus spp.*

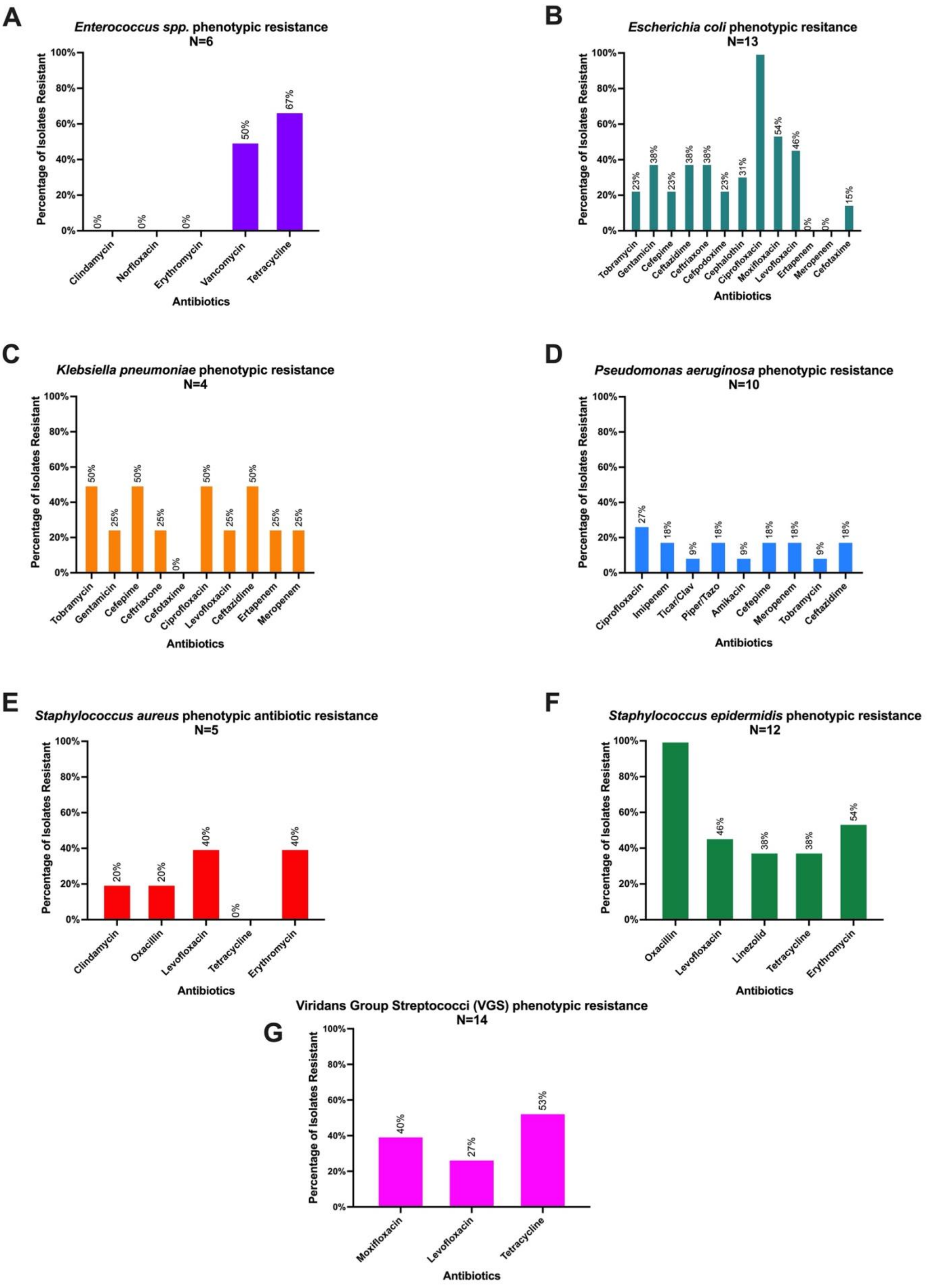
A
Enterococcus spp. phenotypic resistance
N=6
Percentage of Isolates Resistant
100%
80%
60%
40%
20%
0%
0%
0%
0%
50%
67%
Clindamycin
Norfloxacin
Erythromycin
Vancomycin
Tetracycline
Antibiotics
B
Escherichia coli phenotypic resitance
N=13
Percentage of Isolates Resistant
100%
80%
60%
40%
20%
0%
23%
38%
23%
38%
38%
23%
31%
54%
46%
0%
0%
15%
Tobramycin
Gentamicin
Cefepime
Ceftazidime
Ceftriaxone
Cefpodoxime
Cephalothin
Ciprofloxacin
Moxifloxacin
Levofloxacin
Ertapenem
Meropenem
Cefotaxime
Antibiotics
C
Klebsiella pneumoniae phenotypic resistance
N=4
Percentage of Isolates Resistant
100%
80%
60%
40%
20%
0%
50%
25%
50%
25%
0%
50%
25%
50%
25%
25%
Tobramycin
Gentamicin
Cefepime
Ceftriaxone
Cefotaxime
Ciprofloxacin
Levofloxacin
Ceftazidime
Ertapenem
Meropenem
Antibiotics
D
Pseudomonas aeruginosa phenotypic resistance
N=10
Percentage of Isolates Resistant
100%
80%
60%
40%
20%
0%
27%
18%
9%
18%
9%
18%
18%
9%
18%
Ciprofloxacin
Imipenem
Ticar/Clav
Piper/Tazo
Amikacin
Cefepime
Meropenem
Tobramycin
Ceftazidime
Antibiotics
E
Staphylococcus aureus phenotypic antibiotic resistance
N=5
Percentage of Isolates Resistant
100%
80%
60%
40%
20%
0%
20%
20%
40%
0%
40%
Clindamycin
Oxacillin
Levofloxacin
Tetracycline
Erythromycin
Antibiotics
F
Staphylococcus epidermidis phenotypic resistance
N=12
Percentage of Isolates Resistant
100%
80%
60%
40%
20%
0%
46%
38%
38%
54%
Oxacillin
Levofloxacin
Linezolid
Tetracycline
Erythromycin
Antibiotics
G
Viridans Group Streptococci (VGS) phenotypic resistance
N=14
Percentage of Isolates Resistant
100%
80%
60%
40%
20%
0%
40%
27%
53%
Moxifloxacin
Levofloxacin
Tetracycline
Antibiotics

Supplemental Figure 7. Graphical representation of phenotypic antibiotic resistance. The percentage of isolates resistant to various antibiotics for A) *Enterococcus spp.*, B) *Escherichia coli,* C) *Klebsiella pneumoniae,* D) *Pseudomonas aeruginosa,* E) *Staphylococcus aureus*, F) *Staphylococcus epidermidis* and G) Viridians Group Streptococci (VGS). Each graph was made by looking at what antibiotics were tested for sensitivity for each species of interest, and determining what percentage of isolates were resistant to each antibiotic.
